# Case Report: Case of cardiac metastases from melanoma, treated by stereotactic radiotherapy, using a MICRA implant

**DOI:** 10.3389/fcvm.2025.1588106

**Published:** 2025-08-15

**Authors:** Mehdi Belbachir, Nicolas Danthez, David Rotzinger, Antiochos Panagiotis, Etienne Pruvot, Veronique Vallet, Maude Gondre, Gregoire Berthod, Sofia Latifyan, Rita Demicheli, Luis Schiappacasse

**Affiliations:** ^1^ Department of Radiation Oncology, Lausanne University Hospital (CHUV), Lausanne, Switzerland; ^2^ Department of Diagnostic and Interventional Radiology, Lausanne University Hospital (CHUV) and University of Lausanne (UNIL), Lausanne, Switzerland; ^3^ Department of Cardiology, Lausanne University Hospital (CHUV), Lausanne, Switzerland; ^4^ Institut de Radiophysique, Lausanne University Hospital (CHUV), Lausanne, Switzerland; ^5^ Department of Oncology, Valais Hospital, Sion, Switzerland; ^6^ Department of Oncology, Lausanne University Hospital (CHUV), Lausanne, Switzerland

**Keywords:** SBRT, cardiac metastasis, melanoma, case report, radiotherapy, CyberKnife

## Abstract

Metastatic melanomas with prolonged survival are becoming increasingly common. We present the case of a 47-year-old man with melanoma who developed asymptomatic cardiac metastases, whose prognosis depended on their response to either systemic or focal treatment. Consequently, a CyberKnife radiotherapy treatment was performed using a pacemaker for tracking. Instead of using a pacemaker lead, we report here the successful use of a leadless pacemaker (Micra, Medtronic) as a fiducial reference for the tracking, which proved to be reproducible during all sessions. The planning target treatment volume was 161.58 cm^3^. The radiotherapy treatment was well tolerated, and follow-up cardiac CT scans performed at 1 month and 4 months after the treatment showed an approximately 30% reduction in the lesions size. The size reduction was attributed to the focal radiotherapy treatment, as the other metastatic lesions were progressively worsening during the same period. This case report highlights the feasibility of using a leadless pacemaker as a tracking fiducial for the CyberKnife treatment of high-volume cardiac metastatic lesions.

## 1 Introduction

### 1.1 The current state of metastatic melanoma

The incidence of melanoma is approximately 10 per 100,000 in European countries, and is increasing despite a stabilization in mortality rates (1).

Prognosis of patients with stage III and IV melanoma has been changed by immune and targeted therapies, which have demonstrated a significant improvement in survival compared to chemotherapy (1). Despite these advances, the prognosis remains poor.

### 1.2 Cardiac metastasis clinical presentation and treatment options

Primary tumors leading cardiac metastases can be divided into three categories based on their incidence: common tumors with an intermediate rate of cardiac metastases, including stomach, liver, ovary, colon, and rectum carcinomas; less common primary tumors with a high rate of cardiac metastases, such as melanoma, germ cell neoplasms, and malignant thymoma; and common tumors with rare cardiac metastases (2).

Cardiac metastases are often asymptomatic and not easily detected with conventional diagnostic methods. However, advancements in diagnostic modalities have led to a marked increase in the number of patients diagnosed with cardiac metastases (3). Extracardiac malignancies can spread to the heart through four pathways: direct invasion (commonly from mediastinal tumors), hematogenous spread, lymphatic spread, and intracavitary extension (typically via the inferior vena cava) (2).

The reported incidence of cardiac metastases from cutaneous melanoma (CM) varies, ranging from 0.2% to 11.8%. However, these numbers are difficult to interpret, as they are primarily derived from autopsy series (2, 4). Cardiac metastases are most effectively detected via cardiac MRI, which is not routinely performed in patients with metastatic cancer (5, 6).When they occur, cardiac metastases can be challenging to distinguish from other causes of cardiovascular disease. Positron emission tomography (PET) is useful for identifying metabolically active lesions and assessing systemic disease involvement. Computed tomography (CT), on the other hand, is particularly useful for volumetric analysis and precise targeting in local therapies, such as radiotherapy, due to its high spatial resolution (7). The most common symptoms of cardiac metastases include arrhythmia, and signs of heart failure with dyspnea, lower limb edema and chest pain (8).

Patients are typically offered a variety of therapeutic options, including palliative systemic treatments and, in rare cases, surgical excision (4). Radiotherapy has a role as a palliative or ablative treatment. Treating cardiac metastases, however, is challenging due to cardiac motion caused by contractility and respiratory movements (9, 10), plus there is the maximum tolerated dose to the heart and its nearest critical organs.

Cardiac metastases present a technical challenge for radiotherapy due to the continuous motion caused by cardiac contractility and respiration; it compromises dose accuracy, few solutions are available for intrathoracic tracking. Fiducials are often placed invasively, with risks and limitations, previous reports have explored various approaches, but no published studies have demonstrated the use of a leadless pacemaker for this purpose. This case explores a novel tracking method and highlights its implications for broader application.

Furthermore, specific challenges in targeting cardiac metastases include anatomical variability, movement from both respiration and heartbeat, and the proximity of critical organs. While MR-based tracking and breath-hold techniques are evolving (9, 11), real-time tracking using a stable, implantable fiducial remains a reliable approach, particularly when paired with stereotactic radiotherapy platforms such as CyberKnife.

## 2 Case presentation

The patient is a 47-year-old man diagnosed with a stage III melanoma diagnosed in 2017, treated within a clinical trial with ipilimumab and nivolumab. The patient progressed to stage IV in october 2019, and his disease continued to worsen despite immunotherapy. Cardiac metastases were detected on a PET-CT scan in april 2024. Radiotherapy was discussed to treat these lesions during a multidisciplinary tumorboard. Cardiac MRI showed two metastases: a large 53 mm lesion, attached to the lateral wall of the right atrium and extending from its base to its roof, with part of it extending into the superior vena cava; and a second 28 mm lesion, attached to the basal lateral wall of the right ventricle, showing the same characteristics as the first lesion.

The treatment method relied on stereotactic radiotherapy (SBRT) with CyberKnife, Synchrony tracking method with pacemaker probe as fiducial. The patient underwent implantation of a Microport VVI TEO pacemaker (Supplementary Material S1). The baseline examination showed a blood pressure of 119/92 mm Hg, heart rate of 74 bpm, and an oxygen saturation of 96% at ambient air. The initial ECG showed a sinus rhythm at 63 bpm, PR interval <200 ms, QRS 108 ms, a normal axis, peripheral low voltage, a Q wave in DIII, flat T waves in DIII and aVL, and a QTc of 410 ms (overall comparable to the April 2024 ECG). Blood tests revealed no abnormalities. Pacemaker implantation was performed without complications.

**Figure 1:**
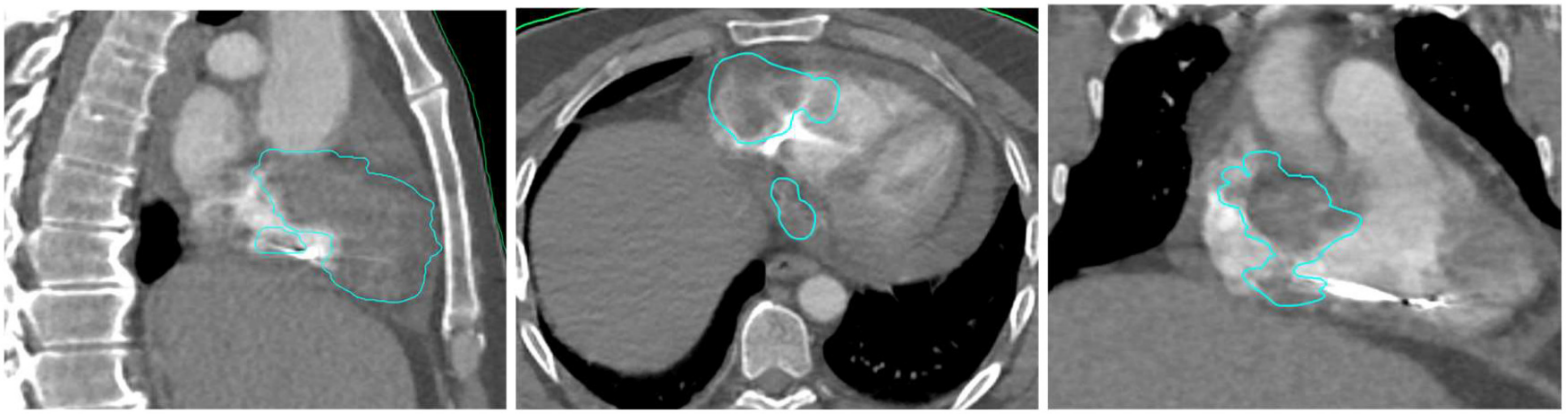
Radiotherapy target delineation.

Due to purulent discharge appearing at the surgical site, the pacemaker device was extracted. A 2-week antibiotic treatment was given for a Staphylococcus aureus infection. A transthoracic echocardiogram did not show any signs of endocarditis or valvular regurgitation. A MICRA pacemaker was then successfully implanted transvenously (Supplementary Material S2), placed directly into the right ventricle without leads, avoiding surgical pocket creation and potentially reducing infection risk. The implantation procedure went with no procedural adverse events and the patient recovered quicly. The workup was repeated with a dedicated cardiac CT scan (Supplementary Material S3), a cardiac MRI (Supplementary Material S4), and an 18-FDG PET scan (Supplementary Material S5). The treatment planning system used was Raystation. Thanks to this multimodal imaging preparation, we were able to delineate a most representative target volume (Figure 1). No dedicated respiratory motion control system was used, as Synchrony tracking compensated for respiratory variation, the cardiac motion was indirectly managed by using the pacemaker as a surrogate, ensuring dose alignment with the tumor volume.The patient was immobilized in the supine position using knee and head cushions and vacuum-locked arms positioned along the body.

**Figure 2:**
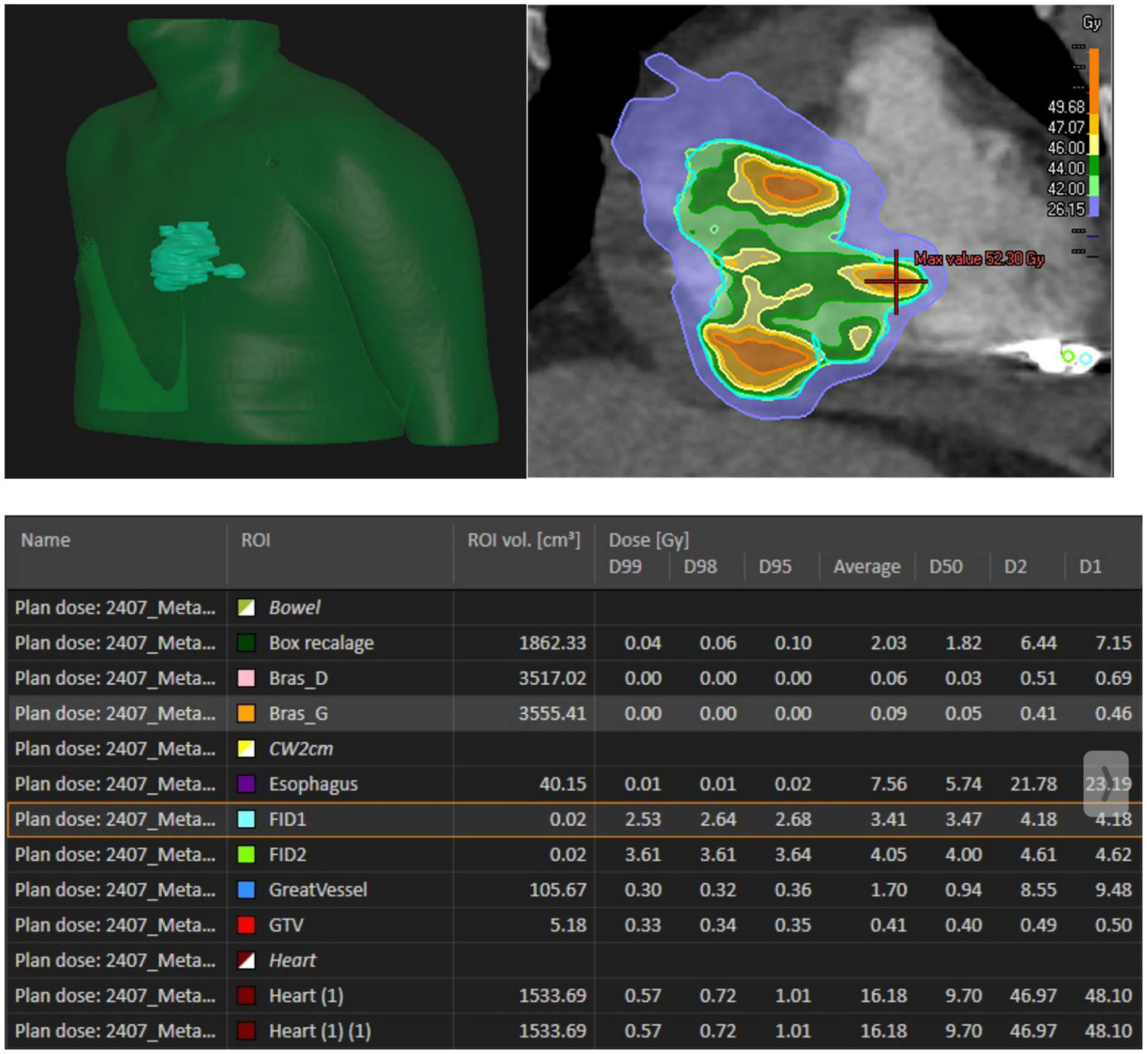
Radiation dose distribution. Bras D: right arm, Bras G: left arm, FID 1: fiducial 1, FID2: fiducial 2.

**Figure 3:**
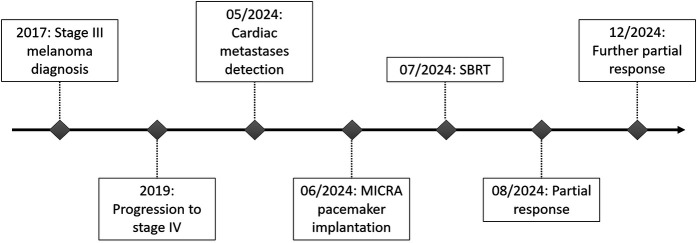
Patient timeline.

The prescribed dose was 42 Gy in 6 fractions (7 Gy per fraction), covering 97.3% of the PTV and 99.82% of the GTV (Supplementary Material S6). Organs at risk (OARs) including lungs, esophagus, liver, and stomach respected accepted dose constraints. However, the heart dose exceeded standard constraints, given that it was the target. Critical organ dose limits and the full dose/volume histogram were reviewed and respected where applicable (Figure 2). The full timeline for the patient is displayed in Figure 3.

**Figure 4:**
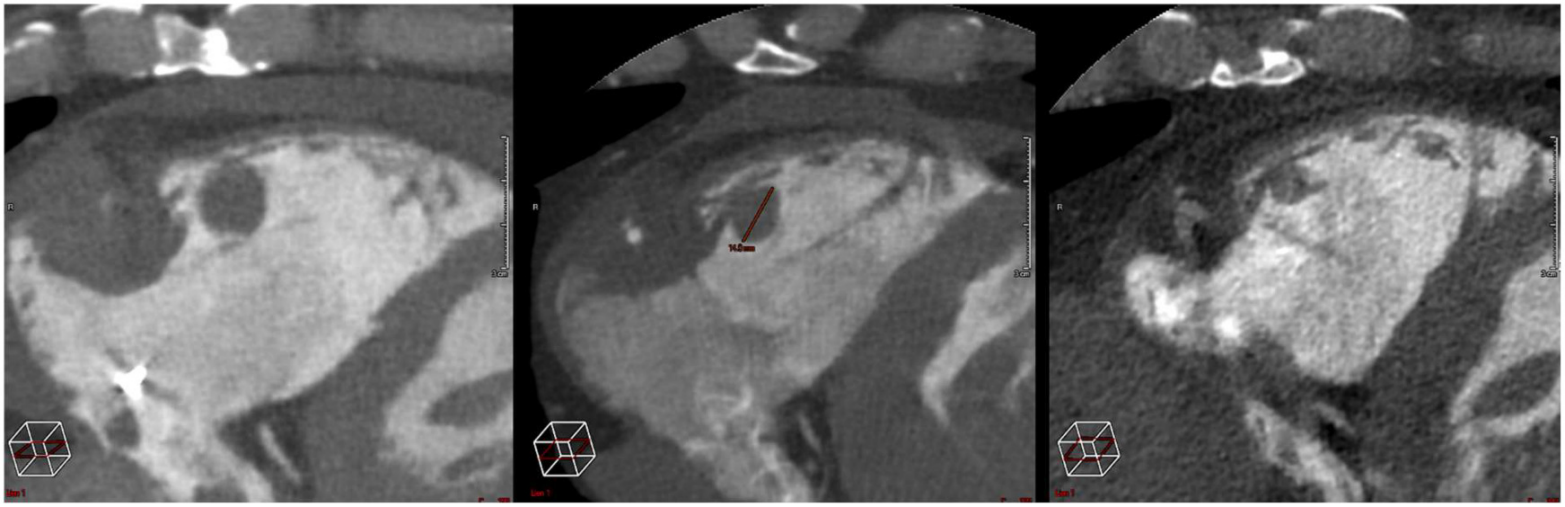
Follow up injected ct scans imaging, from left to right: baseline, 1 month, 4 months.

After the treatment, the patient experienced no cardiovascular events. There were no acute or late clinical toxicities (>3 months). The latest clinical and radiological evaluation, conducted 4 months after the completion of the treatment, included a cardiac CT scan to assess the response. Scans performed at 1 month and 4 months (Figure 4) showed a favorable evolution, with a reduction in size of the treated lesions (Supplementary Material S7).

## 3 Discussion

SBRT (Stereotactic Body Radiation Therapy) for cardiac metastases using CyberKnife and tracking using a MICRA PM rather than a PM lead is, to the best of our knowledge, unprecedented. It has already been established that treating cardiac metastases with SBRT is feasible, demonstrating promising response outcomes (11). While previous studies have used pacing leads, our approach avoids invasive placement and offers high reproducibility, other methods for cardiac tracking include: breath-hold techniques, implanted fiducials, or MR-guided radiotherapy, each presents unique challenges, especially in patients with high cardiac motion or poor compliance (9, 12)

The complexity of this type of treatment lies in the variability of lesion locations; indeed, any of the cardiac chambers can host metastatic lesions, which makes fiducial placement very challenging or even impossible in some cases. Furthermore, such procedures are often invasive, requiring general anesthesia and transesophageal echocardiography. The placement of a leadless PM offers the advantage of being a straightforward and widely performed procedure by electrophysiologists. The Synchrony system has already shown its ability to track PM or defibrillator leads, tracking down the target area according to respiratory motions (13). This setup was mainly made possible by our team's large experience in Stereotactic arrhythmia radioablation for refractory ventricular tachycardia (STAR) (14).

This case demonstrates that a cardiac metastasis, despite its high volume, can be tracked using CyberKnife. Additionally, tracking based on a leadless PM is fully compatible with multiple fractionations, with satisfactory interfraction reproducibility and excellent immediate clinical tolerance.

Local control was also noteworthy. Indeed, while the patient's disease was globally progressing under systemic treatment, the heart lesion decreased by approximately 30% and 60% as seen in PET-CT and cardiac CT scans performed at 1 month and 3 months period, respectively.

Limitations include: the single-case nature of this report, lack of long-term follow-up, and uncertainty regarding the impact of radiation on cardiac function, it may include: arrhythmia, myocardial damage, and late-onset fibrosis, especially for lesions near the conduction system or coronary vessels. Further studies are needed to assess safety, optimal dose constraints, and long-term outcomes.

## 4 Patient perspective

The patient expressed satisfaction with the procedure and outcome and was thankful for the opportunity, he reported no discomfort during the treatment nor notable side effect. He expressed interest in contributing to future case series to help validate this approach.

## Data Availability

The original contributions presented in the study are included in the article/Supplementary Material, further inquiries can be directed to the corresponding author.
