# Supplementary material for: Case Report: Case of cardiac metastases from melanoma, treated by stereotactic radiotherapy, using a MICRA implant
Source: Front Cardiovasc Med. 2025 Aug 15;12:1588106. doi: 10.3389/fcvm.2025.1588106 (PMC12394135; doi:10.3389/fcvm.2025.1588106)
Supplement: Supplementary file 1 [file Datasheet1.pdf]

Supplementary material:

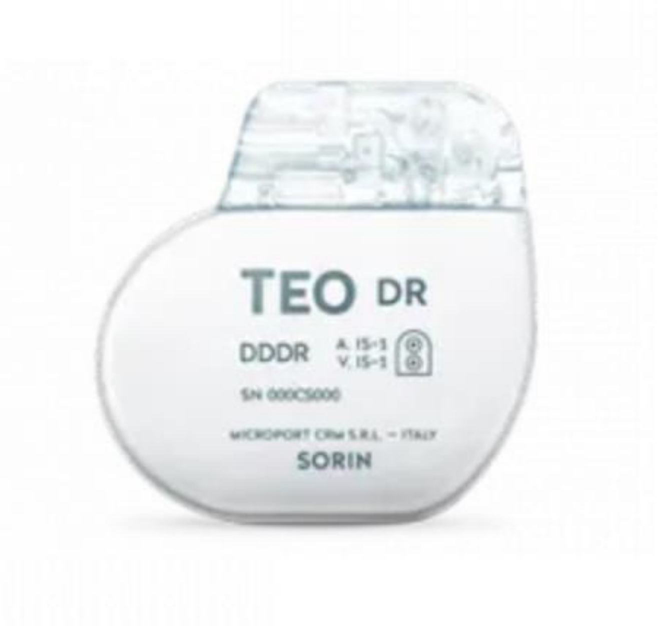

Supplementary Material 1: the initial pacemaker a Micro pacemaker

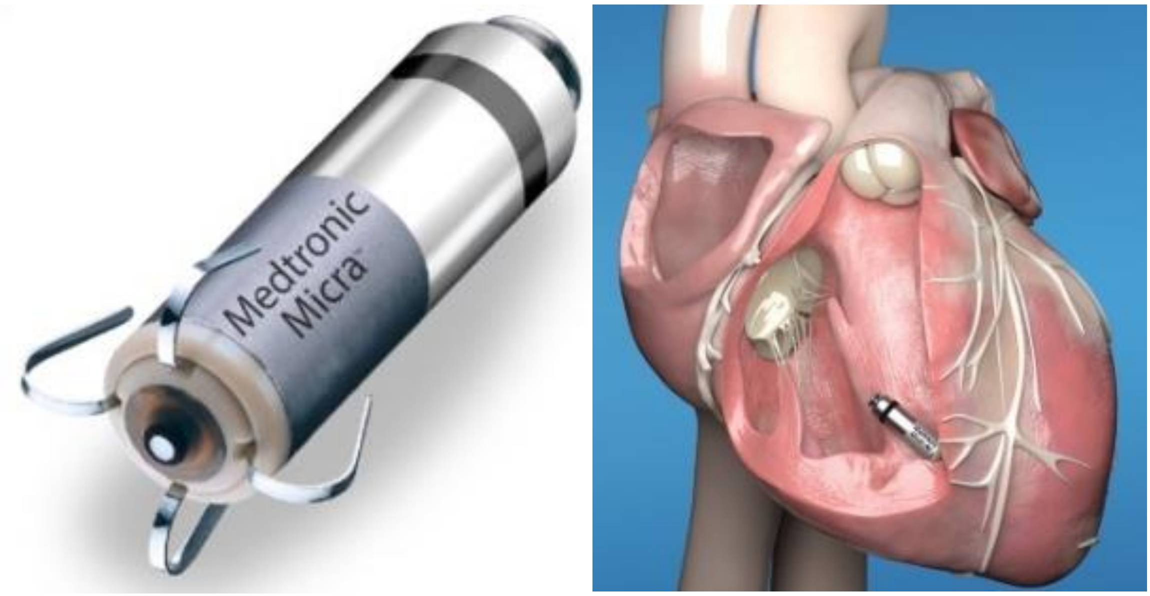

Supplementary Material 2: MICRA pacemaker, used for the actual treatment

Supplementary Material 3: Initial CT reports

<u>Cardiac CT of June 4, 2024:</u>

Description : Several intracardiac lesions centered on the right chambers, associated with known melanoma metastases, are observed, as follows:

- A large hypodense intracardiac mass centered on the inter-atrio-ventricular groove, with invasion of the tricuspid annulus and encasing the right coronary artery, measuring approximately 76 x 56 mm on axial slices and showing significant tissue enhancement (hypermetabolic on PET CT).
- A rounded hypodense lesion of 17 x 15 mm, with enhancement, located near the trabeculae and anterior papillary muscle of the right ventricle, in contact with the mass, and without metabolic activity on the PET CT, suggesting a thrombotic component as the primary hypothesis.
- Two enhancing lesions in the coronary sinus and at the atrio-caval junction, measuring 13 and 11 mm, suggestive of metastases.
- A hypodense enhancing lesion in the right atrial appendage, measuring 14 mm, suspected to be a metastasis.
- Presence of a pseudo-nodular lesion of the interventricular septum, measuring 13 mm, not well-characterized on this exam but better visualized on the MRI of 17/05/2024.

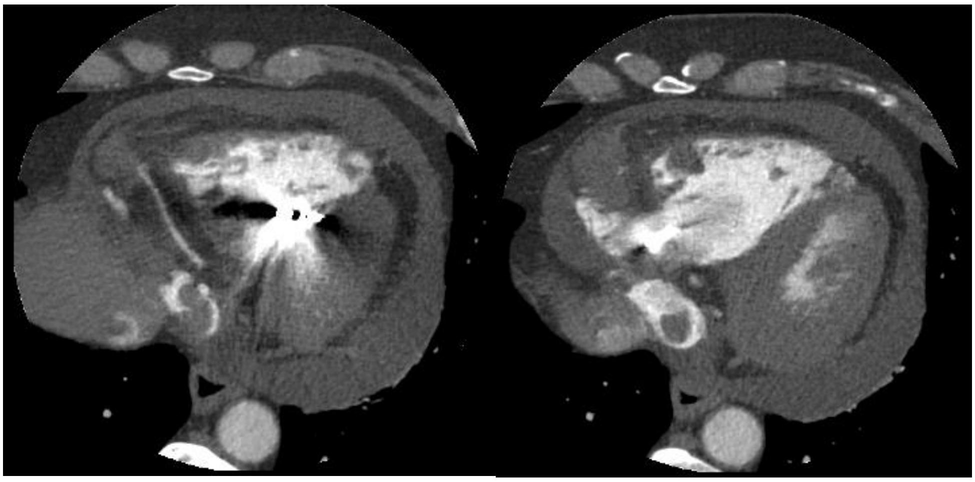

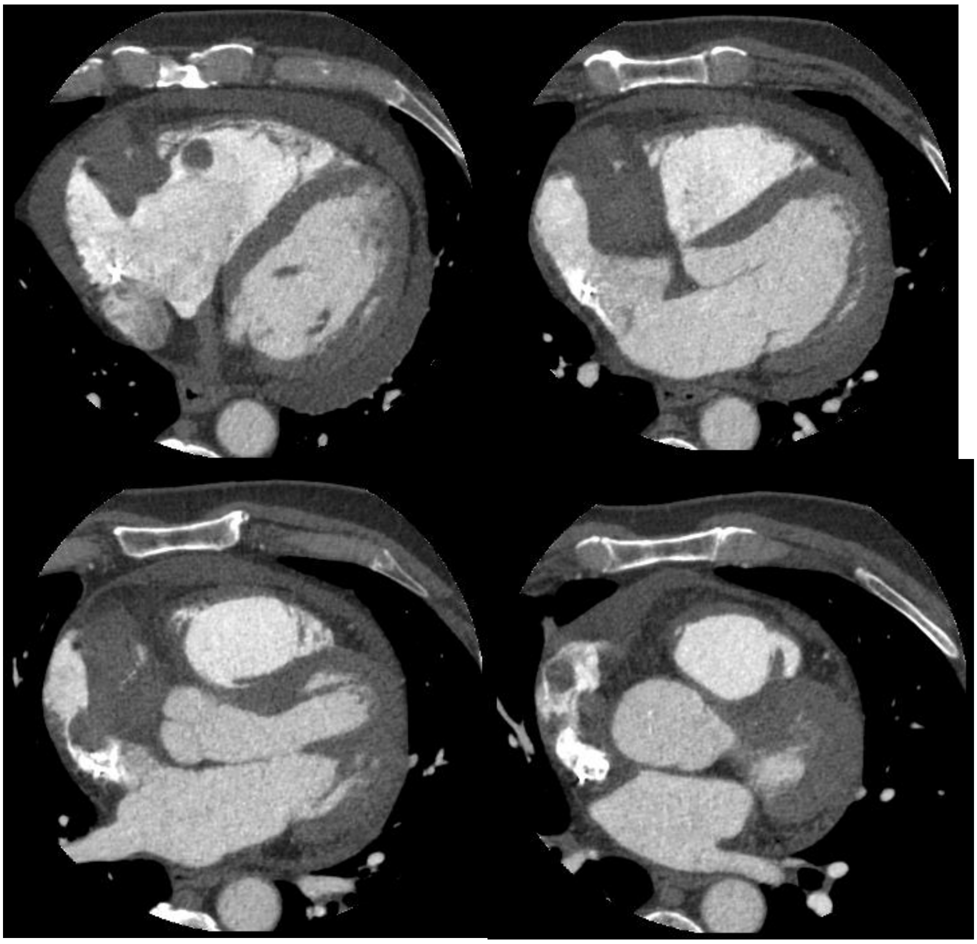

Supplementary Material 4: Heart MRI reports

<u>Cardiac MRI of May 17, 2024:</u>

Description : The large oval mass, measuring 53 x 38 x 51 mm, is located adjacent to the lateral wall of the right atrium, extending from its base to its roof, and showing the extension of a mobile part into the superior vena cava. The course of the right coronary artery runs within the mass. These are characteristic of melanoma metastases

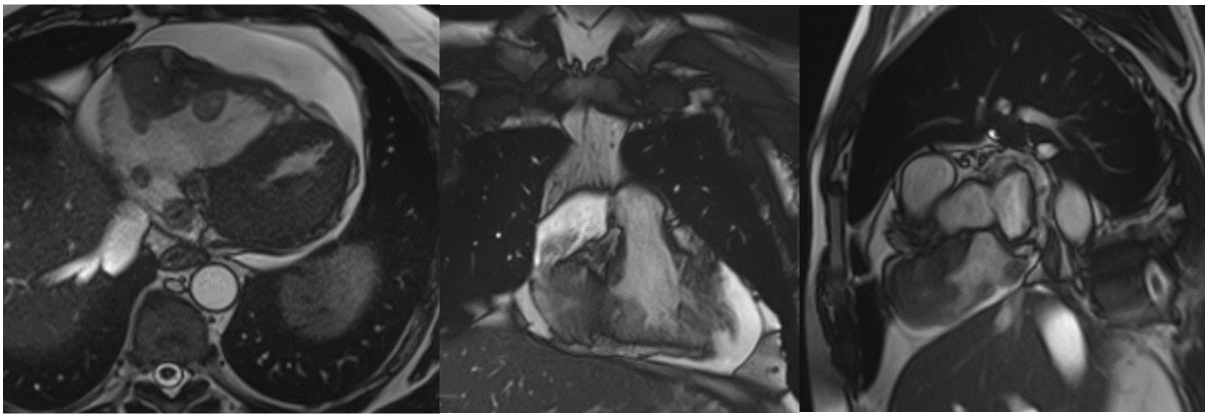

Supplementary Material 5:

18-FDG PET CT of May 31, 2024:

Description: There is hypermetabolism within a large heterogeneous lesion, described on the cardiac CT of 06/04/2024, centered on the inter-atrio-ventricular groove, with invasion of the tricuspid annulus and encasing the right coronary artery, measuring approximately 55 x 67 mm, compatible with a tumor origin. No suspicious ventricular focus; the left ventricular uptake in the lateral and inferior regions observed on the comparative examination remains consistent with physiological uptake.

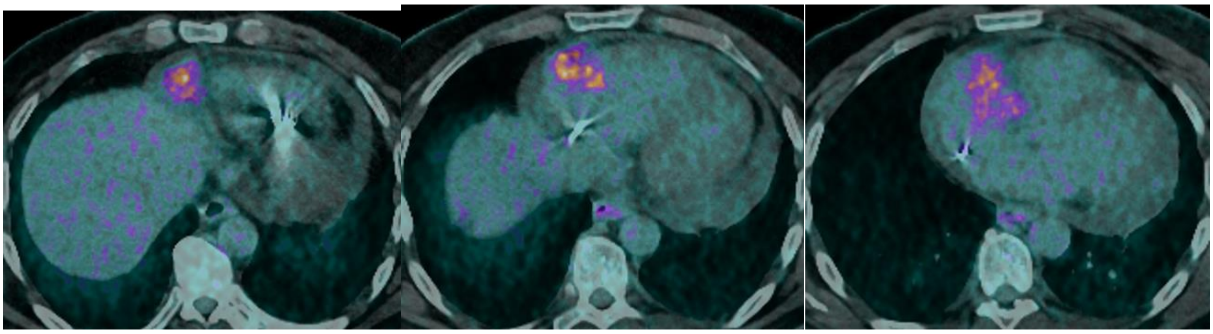

Supplementary Material 6: Radiation therapy prescription

Treatment site •	PTV métas cardiaques	Particle type •	Photons
Prescription type •	Near min dose (D98%)	Treatment technique	Cyberknife
Dose volume [%]		Fractionation fx/day	1
Prescribed dose/fx [Gy] •	7.00	Fractionation days/week	3
Number of fractions •	6	Remark	iso 80 %
Prescribed dose [Gy]	42.00		

Supplementary Material 7 :

Cardiac CT of August 30, 2024:

Findings: Reduction in size of multiple intracardiac lesions centered on the right chambers, related to known melanoma metastases, as follows:

- **Reduction in size of the oval lesion** within the right ventricle, from 18 mm to 15 mm.
- **Reduction in size of the large lesion** at the roof of the right atrium, encasing the right coronary artery without causing stenosis, and invading the tricuspid annulus, from 62 x 40 mm to 48 x 28 mm.
- **Reduction in size of the lesion** in the coronary sinus, from 24 mm to 11 mm.
- **Reduction in size of the lesion** at the superior atrio-caval junction, from 35 mm to 16 mm in its longest axis.
- **Reduction in size of the lesion** at the emergence of the coronary sinus, from 9 mm to 6 mm.

Conclusions :

- Approximately 30% reduction in size of all lesions in the right heart chambers.
- No new suspicious intracardiac lesion identified during the interval.
- Partial regression of the pericardial effusion seen on the comparative, now non-circumferential.

Cardiac CT of December 2, 2024 :

Findings: Continued regression in size of multiple cardiac metastatic lesions, predominantly in the right heart chambers. For example, a nodule in the right ventricle measuring 10 mm (305-1726), compared to 17 mm on June 4, 2024. No new lesions. No stenosis of the right coronary artery despite known neoplastic encasement. Small amount of pericardial effusion.

Conclusion:

Favorable evolution compared to August 2024 regarding the cardiac involvement, with a reduction in size of the known lesions; however, in the acquisition field, there is progression of hepatic and lymph node involvement compared to October 30, 2024.
